# Cytokines in Inflammatory Disease

**DOI:** 10.3390/ijms20236008

**Published:** 2019-11-28

**Authors:** Shinwan Kany, Jan Tilmann Vollrath, Borna Relja

**Affiliations:** 1 Experimental Radiology, Department of Radiology and Nuclear Medicine, Otto von Guericke University Magdeburg, 39120 Magdeburg, Germany; s.kany@uke.de; 2 Department of Cardiology with Emphasis on Electrophysiology, University Heart Centre, University Hospital Hamburg-Eppendorf, 20251 Hamburg, Germany; 3 Department of Trauma, Hand and Reconstructive Surgery, Goethe University, 60590 Frankfurt, Germany; 4 Experimental Radiology, Department of Radiology and Nuclear Medicine, Otto von Guericke University Magdeburg, 39120 Magdeburg, Germany

**Keywords:** inflammation, disease, interleukin

## Abstract

This review aims to briefly discuss a short list of a broad variety of inflammatory cytokines. Numerous studies have implicated that inflammatory cytokines exert important effects with regard to various inflammatory diseases, yet the reports on their specific roles are not always consistent. They can be used as biomarkers to indicate or monitor disease or its progress, and also may serve as clinically applicable parameters for therapies. Yet, their precise role is not always clearly defined. Thus, in this review, we focus on the existing literature dealing with the biology of cytokines interleukin (IL)-6, IL-1, IL-33, tumor necrosis factor-alpha (TNF-α), IL-10, and IL-8. We will briefly focus on the correlations and role of these inflammatory mediators in the genesis of inflammatory impacts (e.g., shock, trauma, immune dysregulation, osteoporosis, and/or critical illness).

## 1. Introduction

The first line of defense against molecules that are either pathogen-derived or endogenous danger signals (or quite often both) has evolved over millions of years. It is composed of players and mediators that are common to most vertebrates and invertebrates, as well as even plants [1]. In general, immunity does not only differentiate between self and not-self but also between dangerous and not dangerous [2]. Inflammation-inducing pathogen-associated molecular patterns (PAMPs) include highly conserved structures [3] such as cytosine-phosphate-guanin motifs [4], heat shock proteins (HSP), peptidoglycans (PGN), and lipopolysaccharides (LPS) [5], while danger-associated molecular patterns (DAMPs) are originally intracellular proteins or nucleic acids normally not found outside the cell, such as chromatin-associated protein high-mobility group box 1 (HMGB1), adenosine triphosphate (ATP), uric acid, deoxyribonucleic acid (DNA), and degraded extracellular matrix-like heparan sulphate and hyaluronan [6,7]. Both PAMPs and DAMPs are recognized via pattern recognition receptors (PRRs) [8,9,10]. Toll-like receptors (TLR), nucleotide-binding oligomerization domain (NOD)-like receptors (NLR) and mannose binding lectin (MBL) are major PRRs implicated in inflammatory pathways [5,11]. Upon their activation, PRR transduces signals intracellularly [12], e.g., via mitogen-activated protein (MAP) kinase signaling pathways to nuclei where diverse transcription factors, such as nuclear factor ‘kappa-light-chain-enhancer’ of activated B-cells (NF-κB) get activated and induce a cellular response [13]. This response often involves the induction of adhesion molecules accelerating inflammation and diapedesis of effector cells of the innate immunity [14]. Furthermore, it leads to the induction, production and the release of pro-inflammatory mediators, including cytokines such as tumor necrosis factor-alpha (TNF-α), interleukin (IL)-1, IL-6 and IL-10, or type 1 interferons (IFNs) [15]. Additionally, those inflammatory mediators themselves can induce, for example, DAMPs to potentiate inflammation [16]. Summarized, the messenger molecules such as cytokines are highly important in the orchestration of the inflammatory response to self- or not-self danger molecules. Meanwhile, the role of the immune system in various inflammatory diseases, traumas, and bone pathologies, such as osteoporosis, osteoarthritis, and rheumatoid arthritis is well described. In this review, we will briefly describe IL-1, IL-6, IL-8, IL-10, and TNF-α cytokines.

## 2. Cytokines

Cytokines are small secreted proteins (<40 kDa), which are produced by nearly every cell to regulate and influence immune response [5]. The release of pro-inflammatory cytokines will lead to activation of immune cells and production as well as the release of further cytokines [17]. Therefore, in the past when the term “cytokine storm” arose, it explained inflammation as a sudden release of cytokines to upregulate an inflammatory process [18]. However, recent research indicates that a simultaneous release of pro- and anti-inflammatory cytokines are mandatory in any immune response [19].

**Figure 1:**
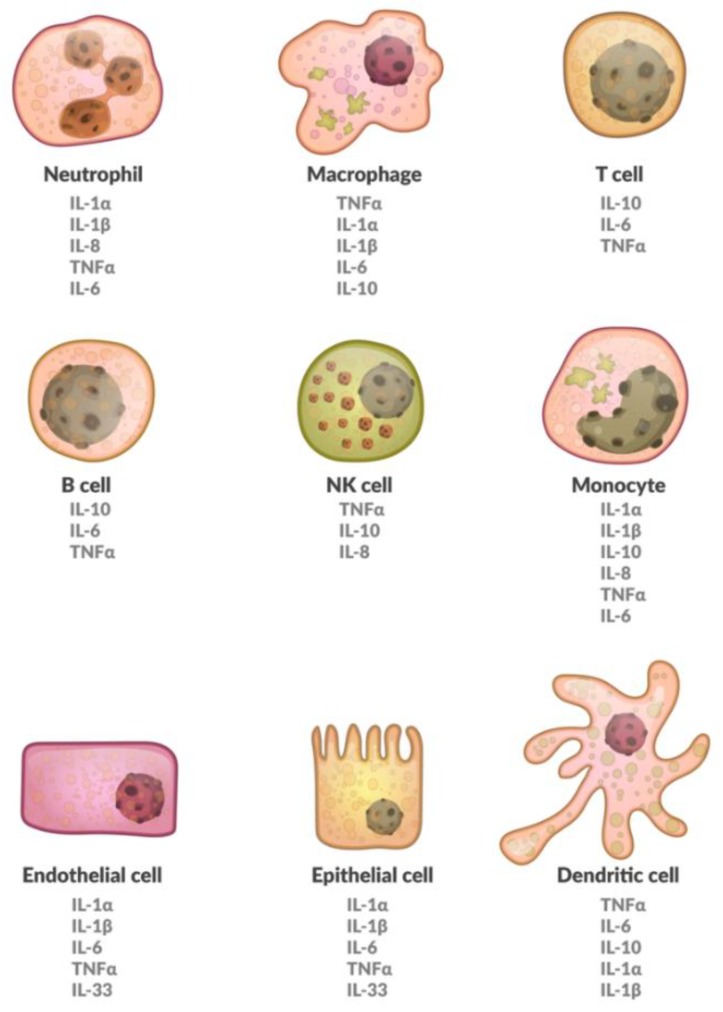
A schematic representation of various cells expressing different cytokines. Interleukin (IL), Natural killer cells (NK), Tumor necrosis factor-alpha (TNF-α).

Cytokines suffer from a somewhat inconsistent nomenclature; they are referred to as interleukins, chemokines, or growth factors among many other names [20]. Cytokines are made up of so-called superfamilies, not necessarily describing common genes, but rather similar structures [21]. Furthermore, different cell populations can produce the same cytokine. The effects of cytokines depend on the targeted cell, making them pleiotropic [20]. Also, different cytokines may have the same effect and are therefore redundant. They may, however, also have a synergistic effect. Finally, they potentially trigger signaling cascades, giving the smallest amounts of protein the chance to be devastating in consequence [22]. A brief overview on various cells expressing different cytokines has been provided in Figure 1. Furthermore, a brief overview of cytokines that are involved in osteoporosis is shown in Table 1.

### 2.1. Interleukin-6

Interleukin-6 has been shown to play important roles in autoimmune diseases, bacterial infections and metabolic side effects have been observed also [23]. It is composed of four α-helices, comparable to other members of the IL-6 family [23]. It is translated as a 184 amino acid long protein that undergoes glycosylation and is secreted by T-cells, monocytes, endothelial cells, and fibroblasts [17]. Interestingly, IL-6 was first described for its effects on adaptive immunity, like promoting cluster of differentiation (CD)4^+^ T-cells via IL-21 production, and promoting T-cell differentiation towards T-helper2 cells (Th2) and Th17 cells [24]. The very first reference was as a B-cell stimulatory factor by the Kishimoto group in 1986 [25]. It has pro- and anti-inflammatory properties, which are described further below. Signaling is achieved via two different mechanisms; one of which is IL-6 binding to its membrane-bound IL-6 receptor (mbIL6R) [26]. This complex subsequently recruits two molecules of membrane-bound glycoprotein (gp) 130, a process that leads to downstream signaling via Janus kinases/signal transducer and activator of transcription (STAT) kinases, phosphoinositide 3-kinase (PI3K), and MAP kinases like p38 [27,28]. A major limitation for a sustainable reaction to IL-6 is the availability of the mbIL6R, which is only expressed on certain cell types, while gp130 is found in almost every cell [29]. This implies that the systemic influence of IL-6 via classic signaling is rather limited [17]. The second mechanism of IL-6 recognition is dependent on the soluble IL-6 receptor (sIL6R), which is expressed via mRNA splicing or proteolysis by a disintegrin and metalloproteinase (ADAM) proteases [17]. Interestingly, ADAM proteases cannot be activated only by other cytokines, such as IL-1β or TNF-α [30], they can be induced by bacterial toxins as well [31]. In the case of sIL6R expression, IL-6 binds to the sIL6R and builds an IL6/sIL6R complex, which in turn activates gp130 on mbIL6R-less cells [32]. This process is termed trans-signaling and is responsible for most of IL-6 inflammation-inducing capabilities [23]. Currently, similar to the C-reactive protein (CRP), IL-6 is used to “monitor” inflammation levels in patients with cancer, infection, or autoimmune diseases [33,34]. The reason for using IL-6 as a biomarker is its central role in activating and maintaining the inflammatory response. For instance, the clinical quantification of IL-6 is a strong predictor for mortality in pancreatic and cardiovascular disease [35,36]. However, unfortunately, its anti-inflammatory properties, further described below, are so far neglected in clinical practice. While early inflammation is dominated by neutrophils, later states of inflammation are dominated by monocytes. IL-6 is essential in this so-called leukocyte switch [37]. Subsequently, it reduces neutrophil recruitment via suppression of chemokines attracting polymorphonuclear leukocytes (PMNL), like the chemokine (C-X-C motif) ligand (CXCL)1 and CXCL8 (IL-8), while upregulating monocyte attracting chemokines CC-chemokine ligand (CCL)2/monocyte chemotactic protein (MCP)-1 and CCL8/MCP-2 in vitro and in vivo [37]. Furthermore, cell adhesion molecules like vascular cell adhesion molecule (VCAM) 1, intercellular cell adhesion molecules (ICAM) and E-selectin are upregulated by IL-6 in a fever range mice model [38]. In models with gp130 knockout mice, the ability of IL-6 to enhance macrophage-colony stimulating factor (M-CSF) receptor expression, thereby accelerating monocyte differentiation to macrophages, was linked to its gp130 MAP kinase pathway [39]. In a *Staphylococcus epidermidis* induced peritoneal inflammation mice model, IL-6 was mandatory for the recruitment of T-cells [40]. Interestingly, classic signaling of IL-6 is needed for regenerative and protective processes in the body. For instance, in inflammatory disease mice models and diverse *knockout* mice models, IL-6 was essential to liver regeneration, gut barrier repair, and suppression of inflammation in the kidney and pancreas [41,42,43].

In clinical practice, the first association of IL-6 with cardiovascular disease and cancer was found in 1990 [44]. Enhanced levels of IL-6 were found in three patients with cardiac myxomas and removal of the tumor abolished the IL-6 levels [44]. In fact, increased pretreatment levels of IL-6 can be a predictor of survival in head and neck cancer [45]. Yet, it often remains unclear if IL-6 is only correlative to cancer or rather essential in cancer genesis. A study by Zhang et al. demonstrated that escalated levels of IL-6R in sera from nasopharyngeal carcinoma (NPC) patients are not just correlative [46]. The cytokine serves as a catalyst for the malignant transformation of Epstein–Barr infected nasopharyngeal cells to cancerous cells in vitro via STAT kinases [46].

Osteoporosis is a common disease in the aging population and studies have shown that IL-6 is potentially implicated in its pathogenesis [47]. IL-6 stimulates bone resorption. Several studies have examined the association between IL-6 gene polymorphisms and bone mineral density [47,48,49].

Another prominent use of IL-6 as a biomarker is in sepsis or after major trauma. Studies in the nineties demonstrated 1000-fold increased IL-6 levels in septic patients and correlation with the gravity of organ failure [50]. Likewise, the detection of IL-6 is correlative to invasiveness and duration of surgery [51]. Levels of IL-6 after trauma usually do not reach those of septic patients [52]. Unlike CRP, IL-6 can also help to distinguish infection from fever of unknown origin in pediatric practice [53]. Several studies confirm a predictive value of IL-6 for mortality and organ dysfunction in sepsis or after major trauma [54,55]. While IL-6 has undoubted prognostic value in early inflammation, clinical use has not seen any breakthroughs. Many physicians prefer a combination of clinical presentation, white blood count, CRP levels, and fever measurement over the expensive IL-6 determination [52].

### 2.2. Interleukin 1 Family

Interleukin-1α and IL-1β were the first cytokines to be discovered in 1974 by Charles A. Dinarello, and since then, they have been greatly studied [56]. In this review, we will focus on the following members of the IL-1 family: IL-1α, IL-1β, and IL-33.

Interleukin-1α and IL-1β are encoded by different genes but can be bound by the same IL-1 receptor (IL-1R) [56]. While IL-1α has a higher affinity for IL1-R1, IL-1β has a higher affinity for the soluble IL-1R2 [57]. Both are translated as 31 kDa precursor protein and cleaved into smaller 17 kDa forms, albeit with different amino acid sequences [58].

The IL-1α precursor is usually found in intracellular space, as well as constitutively in many cell types including hepatocytes, nephrotic epithelium, endothelium, and epithelial cells of the gastro-digestive tract [59].

Even in cases of severe infection, relatively low concentrations are found in extracellular space [60]. Upon stimuli such as oxidative stress or cytokine exposure, e.g., other IL-1 family cytokines, the expression of the IL-1α mRNA is inducible [61]. Nevertheless, it is not clear if post-translational modifications are needed for IL-1α to become active. In contrast to IL-1β and IL-33, the precursor form of IL-1α and recombinant human mature IL-1α have the same biological activity in inducing IL-6 and TNF-α in human peripheral blood mononuclear cells (PBMCs) and lung cancer cells [62]. Nevertheless, the secretion of IL-1α protein is well regulated. During apoptosis, cytosolic IL-1α translocates to the nucleus and binds firmly to chromatin [63], while during necrosis, it becomes released from the nucleus into the local tissue upon degradation of the cell membrane [63]. This exemplifies the properties of IL-1α as an alarmin. Whereas the release of IL-1α during the process of necrosis is explained by the loss of plasma membrane stability, the leakage of IL-1α in “healthy” cells is induced via pyroptosis [64]. This is a process of the so-called inflammation-induced apoptosis, which leads to enhanced cell membrane permeability through the formation of an inflammasome complex in an, e.g., caspase-1-dependent mechanism [64]. Caspase-1 *knock-out* mice displayed significantly less IL-1α protein release by monocytes upon their stimulation with LPS and ATP as compared to the *wild-type* mice [65]. The soluble decoy receptor IL-1R2 functions as a receptor in plasma, and limits spreading of IL-1α, thereby reducing its signaling and restraining inflammation [66]. Another unique trait among the IL-1 family is that the pro-IL-1α in its full length is implemented in the cell membrane in case of inflammation, and can operate as a fully active membrane-bound cytokine [57].

The primary sources of IL-1β are hematopoietic cells like monocytes, macrophages such as microglia or Kupffer cells and dendritic cells upon activation of PRR by PAMP or DAMP [21]. Furthermore, alpha cells of pancreas secrete IL-1β, and this can be studied in diabetic and obese patients [67]. Recent trials imply the contribution of epithelium and endothelial IL-1β to cardiovascular disease [68].

In contrast to IL-1α, IL-1β precursor is not biologically active, as its activation requires a proteolytic step by the IL-1β converting enzyme, e.g., caspase-1 within the multiprotein inflammasome complex [69]. Upon activation of myeloid differentiation primary response (MyD)88 in case of hypoxia, complement activation, or even IL-1β itself, pro-IL-1β mRNA is induced [21,70]. The translation occurs in the cytosol, however, it is discussed if a second signal is required for pro-IL-1β to be cleaved to IL-1β, and thereby activated [71]. Second signals can include DAMP or alarmin molecules, such as ATP, which binds to P2 × 7 receptors, thereby providing a signal to open potassium channels lowering intracellular potassium levels [72]. In consequence, the formation of NOD-like receptor (NLR) sensor molecule, such as NOD-, leucine-rich repeat (LRR)- and pyrin domaine-containing protein (NLRP)3 [73], and apoptosis-associated speck-like protein containing a caspase activation and recruitment domain (ASC) to the inflammasome complex occurs [21]. As a result, pro-caspase-1 is activated into caspase-1 and cleaves, e.g., pro-IL-1β or pro-IL-18 cytokine precursors to their active forms, thereby initiating or enhancing the pro-inflammatory response [74].

In the last decades huge advances have been made in understanding the role of inflammasomes in the pathogenesis of infectious, autoinflammatory and autoimmune diseases. Familial cold autoinflammatory syndrome (FCAS), Muckle–Wells syndrome (MWS) and neonatal-onset multisystem inflammatory disorder (NOMID, also known as chronic infantile neurologic, cutaneous, and articular (CINCA) syndrome) have been shown to be caused by gain of function mutations in the NLPR3 gene encoding for cryopyrin, leading to increased caspase-1 and IL-1β activity [75,76,77,78]. Due to their similar etiology these diseases are today recognized as a group of diseases named pryopyrin-associated periodic syndrome (CAPS). Current treatment of CAPS is successfully and safely based upon three different medications named rilonacept (captures IL-1β as a decoy receptor), anakinra (IL-1R antagonist), and canakinumab (monoclonal antibody against IL-1β) [79,80]. Regarding the role of NRLP3 in the development of atherosclerosis Duewell et al. reported decreased development of atherosclerotic lesions in mice lacking inflammasome related molecules NLRP3, ASC or IL-1α/β and showed that cholesterol crystals as a possible DAMP strongly activated NLRP3 inflammasomes in macrophages [81]. Further studies in similar mouse models as well showed decreased severity of atherosclerosis in mice lacking NLRP3, caspase-1 or IL-1β [82,83,84], while in another study NLRP3 inflammasomes were not critically implicated in atherosclerosis progression [85]. Furthermore, the NLPR3 inflammasome is activated by oxidized low-density lipoprotein (LDL) and high levels of triglyceride, both being major risk factors for atherosclerosis [81,86,87].

The role of inflammasomes in the pathogenesis of rheumatoid arthritis has been studied extensively in the past decades in humans as well as in specific animal models. Guo et al. recently showed that the NLRP3 inflammasome was highly activated in synovia from patients with rheumatoid arthritis and in an animal model with collagen-induced arthritis (CIA) in mice [88]. Furthermore, treatment with MCC950, a selective NLRP3 inhibitor, in the animal model resulted in significantly less severe joints inflammation and bone destruction [88]. In a study by Ippagunta et al. the authors investigated the role of different components of the NLRP3 inflammasome and showed that NLRP3^(−/−)^ and caspase-1^(−/−)^ mice were predisposed to collagen-induced arthritis while ASC^(−/−)^ mice were protected from arthritis [89]. Another study by Joosten et al. investigated the role of caspase-1, the downstream effector of inflammasomes, in the development of rheumatoid arthritis and obtained conflictive results showing no effect of caspase 1 deficiency in a model of acute (neutrophil-dominated) arthritis but reduced joint inflammation and cartilage destruction in a mouse model of chronic arthritis [90]. The crucial role of NRP3 inflammasomes in the development of rheumatoid arthritis was investigated by Vande Walle et al. showing that knock out of A20, a rheumatoid arthritis susceptibility gene, in mice led to increased expression of NLRP3 and pro-IL-1β genes and resulted in induction of NLRP3 inflammasome-mediated caspase-1 activation, pyroptosis, and IL-1β secretion [91].

Furthermore, deletion of NLRP3, caspase-1 and the interleukin-1 receptor markedly protected against rheumatoid-arthritis-associated inflammation and cartilage destruction in A20*^myel-KO^* mice and the authors depicted A20 as a novel negative regulator of NLRP3 inflammasome activation in rheumatoid arthritis [91]. Patients with active rheumatoid arthritis have higher intracellular levels of NLRP3 inflammasome components (including NLRP3, ASC, active caspase-1, and pro-IL-1β) as well as increased secretion of IL-1β [92] and monocytes from patients with rheumatoid arthritis show increased IL-1β production mediated by activation of NLRP3 inflammasome [93]. Shin et al. investigated the role of human umbilical cord blood-derived mesenchymal stem cells (hUCB-MSCs) as a treatment for rheumatoid arthritis in mice with collagen-induced arthritis (CIA) and observed a reduced severity of CIA mediated by a downregulation of the NRLP3 inflammasome [94].

While a huge part of current and past research focused on NLRP3 inflammasomes, it could be shown that the G allele of a polymorphism (rs878329) in the NLRP1 promoter in the Chinese population up-regulates gene transcription and puts patients at risk for developing rheumatoid arthritis [95]. Treatment of CIA mice with BVT-2733, a selective inhibitor of 11β-hydroxysteroid dehydrogenase 1, attenuated arthritis severity by inhibition of the NF-κB and NLRP1 inflammasome signaling pathways [96]. Investigation of treatment with P2X4 antisense oligonucleotide (asODN) in the same CIA model indicated significantly reduced synovial inflammation and joint destruction by inhibition of NRLP1 inflammasome as the underlying mechanism [97].

Recently, mutations in the NLRP1 gene were shown to cause a novel autoinflammatory disorder that the authors proposed to call NAIAD (NLRP1-associated autoinflammation with arthritis and dyskeratosis) which causes arthritis and dyskeratosis [98]. Unfortunately, there are only a few studies analyzing the importance of inflammasomes in the pathogenesis of osteoporosis. IL-18BP, the natural antagonist of proinflammatory IL-18, was shown to be reduced in osteoporotic women [99]. Animal experiments from the same group showed that mIL-18BPd enhances osteoblast differentiation and inhibits the activation of NLRP3 inflammasome and caspase-1 in vitro [99]. In vivo mIL-18BPd treatment restored trabecular microarchitecture, preserved cortical bone parameters and reduced osteoclastogenesis [99].

Xu et al. investigated melatonin treatment in ovariectomized C57BL/6J mice and demonstrated that melatonin improved osteoporosis and impaired osteogenic differentiation potential by suppressing activation of the NLRP3 inflammasome via mediating the wingless-related integration site (Wnt)/β-catenin pathway [100]. Humanized mice carrying an NLRP3 mutation (D305N/D305N mice) developed arthritis and osteoporosis shown by increased radiolucency and thinner cortices in all bones of the lower hindlimb compared to control animals [101]. Kim et al. investigated auranofin, a gold-based compound approved in 1975 for the treatment of rheumatic diseases and found that auranofin suppresses inflammasome mediated IL-1β secretion in mouse bone marrow-derived macrophages (BMDMs) and J774.A1 cells [102]. Furthermore, administration of auranofin in ovariectomized mice led to recovery of bone mass [102].

In in vitro studies with human mesenchymal stem cells (MSCs), activation of NLRP3 inflammasome by lipopolysaccharide and palmitic acid (LPS/PA) treatment led to increased adipogenesis of MSCs and suppressed osteogenesis [103]. The role of inflammasomes in the pathogenesis of age-related diseases especially of the eyes (e.g., glaucoma or age-related macula degeneration) has been extensively studied in the past years and is reviewed profoundly elsewhere. In a mouse model of acute glaucoma the role of HMGB1 has been investigated and it was shown that HMGB1 activates the canonical NLRP3 and non-canonical caspase-8 inflammasomes and production of IL-1β during acute glaucoma development [104]. In a previous study by the same group was shown that inhibition of caspase-8 activation significantly attenuates retinal ganglion cell death by down-regulating the activation of NLRP1 and NLRP3 [105].

Age-related macular degeneration (AMD) is the leading cause of central vision loss worldwide [106] and huge progress has been made in the last decades to understand the role of inflammasomes in the pathogenesis of the disease. Doyle et al. showed that drusen, which are the major pathological hallmark of AMD, isolated from donor AMD eyes activate the NLRP3 inflammasome leading to secretion of IL-1β and IL-18 [107]. Interestingly in a mouse model of wet AMD in NLRP3^(−/−)^ mice laser-induced choroidal neovascularization (CNV) was exacerbated so the authors concluded that NLRP3 and IL-18 might have a protective role in the progression of AMD [107]. The latest findings with regard to the connection between inflammasomes and AMD are thoroughly reviewed elsewhere [108,109]. Trauma is one of the leading causes for death worldwide and although it is indisputable that trauma-injury is closely associated with inflammasomes, there is no clear hypothesis whether the activation of inflammasomes is harmful or beneficial after trauma.

In an ex vivo in vitro experiment with LPS stimulation of CD14^+^-isolated monocytes from trauma patients (TP), gene expression of NLRP1 was markedly reduced compared to healthy controls [110]. Furthermore, transfected monocytes from TP, which expressed the lacking components, were recovered in their LPS-induced IL-1β release, and thus, the authors concluded that lacking NLRP1 is responsible for the suppressed monocyte activity after trauma [110]. NLRP1 has been shown to be an important component of the innate central nervous system inflammatory response after traumatic brain injury (TBI) as its neutralization reduced the innate immune response and improved histopathology after TBI in a mouse model [111]. Furthermore, the NLRP1 inflammasome was found to cause lung injury in a mouse model while lung damage was rather caused by pyroptosis of resident lung macrophages and not by caspase-1 or IL-1β [112]. NLRP1^−/−^ mice were protected from these detrimental effects, indicating the pivotal role of NLRP1 in lung injury [112].

Recent investigations regarding inflammasome proteins as potential biomarker for TBI determined that apoptosis-associated speck-like protein containing a caspase recruitment domain (ASC) in serum and cerebral spinal fluid (CSF) as well as IL-18 in CSF are promising biomarkers of TBI pathology [113]. Moreover, higher protein levels of ASC were consistent with poorer outcomes after TBI [113]. Zhang et al. reported that genetic variations in the NLRP3-gene predict the development of sepsis and multi organ dysfunction syndrome (MODS) in trauma patients [114]. Continuous injury caused by mechanical ventilation, which is common in severely injured trauma patients, is supposed to be mediated by an increase in serum levels of DAMP (e.g., ATP or reactive oxygen species (ROS)), followed by activation of the NLRP3 inflammasome [115,116,117]. To investigate the underlying mechanism of why up to 30% of patients with TBI develop acute lung injury (ALI) or acute respiratory distress syndrome (ARDS), Kerr et al. studied extracellular vesicle (EV)-mediated inflammasome signaling in male C57BL/6 mice [118].

TBI leads to the release of EVs containing inflammasome proteins into serum that target the lung to cause ALI and administration of a blocker of EV uptake (enoxaparin) or monoclonal antibody against ASC improved ALI scores, thus, the authors concluded that neural-respiratory-inflammasome axis is an important part of the innate inflammatory response in lung pathology after TBI [118]. In an animal model of TBI, resveratrol was indicated to attenuate the inflammatory response and relieve TBI by reducing ROS production and inhibiting NLRP3 activation [119]. In burn-injured mice blocking of caspase-1, the downstream effectors of inflammasomes, caused significantly higher mortality, thus, Osuka et al. concluded that inflammasome activation plays a protective role in the host response to severe injury [120]. In contrast, treatment with MCC950, an inhibitor of the NLRP3 inflammasome, led to a better neurological outcome after TBI by alleviating brain edema, reducing lesion volume, and improving long-term motor and cognitive functions in a mouse model with TBI [121].

Inhibition of the NLRP3 inflammasome by treatment with BAY 11-7082 or A438079 alleviated the severity of spinal cord damage and improved neurological recovery after in a mouse model of spinal cord injury [122]. Another recent study showed protective effects in cholestatic liver injury and liver fibrosis by blocking NLRP3 inflammasome activation by treatment with MCC950 [123]. As these are still preclinical studies the value for clinical treatment has to be investigated intensively. In a rat model of subarachnoid hemorrhage minocycline protected against NLRP3 inflammasome-induced inflammation and p53-associated apoptosis, and therefore, the authors concluded that treatment with minocycline treatment may exhibit important clinical potentials in the management of subarachnoid hemorrhage [124]. Denes et al. investigated the role of NLRC4 and AIM2 in a rodent model of stroke and showed that that ischemic brain injury has been reduced in ASC^−/−^ and NLRC4^−/−^ mice without seeing such protective effects, in mice deficient for NLRP3 [125]. Although huge advances have already been made in recent decades, the specific role of the inflammasome in the development of several diseases and therapeutic options still has to be investigated intensively in future.

As the biological activities of both IL-1α and IL-1β are rather similar, this review will refer to them as IL-1 in the following paragraph. Models with IL-1 deficient mice displayed no difference to control mice in terms of growth, homeostasis or fertility, however, they were rather prone to bacterial, mycotic and protozoa infections [21,126]. The ability of IL-1 to stimulate synthesis of inducible nitric oxide synthase (iNOS), Cyclooxygenase (COX)-2 and phospholipase (PL)A2 results in enhanced production of nitric oxide (NO), platelet activating factor as well as prostaglandin (PG)E2 in ex vivo in vitro analyses of chondrocytes from patients with osteoarthritis [127]. Accordingly, the patients in an inflammatory state experienced vasodilation and hypotension, fever and heightened pain sensitivity [128]. To raise systemic dissemination and infiltration of immune cells, chemokine production is upregulated as well as expression of ICAM-1 and VCAM-1 in mesenchymal stem cell models in vitro [129].

Another trait is the augmented permeability of the intestinal barrier and a blood-brain barrier to simplify neutrophil recruitment in these compartments as observed in in vitro models with Caco2-monolayers [130]. A mice model with LPS challenge revealed the angiogenic potential of IL-1 to contribute to blood vessel formation under hypoxic conditions [131]. However, modulation of lymphocytic response like B-cell proliferation is strictly seen as an effect of IL-6 that is inducted by IL-1 as seen in an animal model with either IL-6 or IL-1 knockout mice [132]. In fact, 1 ng/kg bodyweight IL-1 is enough to ensure high systemic levels of IL-6 in mice [21]. Thus, this stresses the important role of IL-1 in disease rather than in healthy individuals. The diagnostic value of IL-1 is rather limited due to its half-life of around 10 min [133]. However, some clinical studies determined IL-1 levels in sera via ELISA. One of those is a work by a Turkish group in neonatal sepsis (*n* = 50) that revealed significantly enhanced levels of IL-1 in septic patients [134]. Another study displayed significant levels of IL-1 in sera of malaria patients compared to control in a cohort of 60 patients [135]. Interestingly, IL1-Ra was not only significantly increased in patients with septic shock on admission day and day 28 but was also a predictor of mortality [136].

Emerging evidence highlights the role of these inflammatory cytokines in the regulation of bone homeostasis. Chronic inflammation is often characterized by an imbalance between bone formation and bone resorption. Here, a net prevalence of osteoclastogenesis has been described, which is an important determinant of bone loss in rheumatic diseases [137]. Yet, the totality of evidence is limited and provides no clear indication of which cytokine is the most important for bone biology. The link between osteoclasts and pro-inflammatory cytokines, especially IL-1, provides an explanation for the association between inflammation and osteoporosis. For inflammatory diseases, bisphosphonates may be chosen as therapy, however specific medications such as denosumab, IL-1 receptor antagonists, or TNF-α antibodies are targeted treatment strategies for osteoporosis secondary to inflammation [138].

In a meta-analysis to examine the efficacy and safety of denosumab in postmenopausal women with osteoporosis by Gu et al., adverse events between verum and placebo group were similar (pooled odds ratio = 1.04, *p* = 0.625) [139]. In a large cohort study Choi et al. observed comparable clinical safety and effectiveness with regard to the risk of serious infection, cardiovascular disease, and osteoporosis fracture within 365 days after initiation of medications between denosumab and zolendronic acid (an established standard of therapy) [140]. Kullenberg et al. investigated the safety of treatment with anakinra, a IL-1 receptor antagonist, in 43 patients for up to five years and observed 24 serious AEs (SAEs), all of which resolved during the study period, in 14 patients with the most common SAEs being infections such as pneumonia and gastroenteritis [141]. The authors concluded treatment with anakinra of patients with severe CAPS for up to 5 years was safe and well tolerated, both in pediatric and adult patients [141]. To assess the safety of treatment with TNF-α antibodies is difficult due to the many different antibodies which are authorized for treatment of e.g., rheumatoid arthritis. Hernández et al. observed a reasonable safety profile for TNF-α antibodies and argue that the benefits far outweigh the possible risk of adverse events [142]. There are several studies and meta-analysis dealing with these TNF-α antibodies and which are extensively reviewed elsewhere.

Interleukin-33 is the newest member of the IL-1 family and located on chromosome 9 [143]. Human IL-33 is located in the cell nucleus but is also found outside the cell as an alarmin [144]. Furthermore, it is synthesized as a 31 kDa protein [143]. The main sources of IL-33 are non-hematopoietic cells such as endothelial and epithelial cells [145]. IL-33 is a ligand to orphan receptor ST2 [146] of the TLR/IL1R superfamily of receptors, thereby potentially activating canonical NF-κB pathway via MyD88 [147]. Nevertheless, it was first described as a nuclear factor from high endothelial venules (NF-HEV) [148]. The name accurately describes its properties being both, a membrane receptor ligand and a nuclear factor for transcription [144]. Furthermore, distinct to other members of the IL-1 family such as IL-1β or IL-18, n-terminal end of IL-33 does not necessitate processing to be active [143]. Nonetheless, IL-33 lacks a secretory sequence for conventional pathways to be secreted into extracellular space [149]. One would suspect necrosis as a primary form causing its release, however, in vivo and in vitro models indicate that living cells secrete IL-33 as well [150]. Recent research of inflammation models like post-viral mice with chronic lung disease and in patients with chronic lung disease indicate that extracellular ATP may play a role in IL-33 expression [151]. Ex vivo analysis of airway basal cells of the mice revealed significant IL-33 secretion upon ATP exposure [151].

Its biological impact is associated with the type 2 immune response, mainly reliant on the activation of Th2 cells, eosinophils, mast cells, basophils and group 2 innate lymphoid cells (ILC-2) [143,149]. These cell populations express ST2 and show the importance of IL-33 in allergic and autoimmune disease [145,152]. For instance, chronic exposure of cigarette smoke to mice leads to enhanced systemic IL-33 levels [153]. Additionally, IL-33 skews T-cells toward Th2 differentiation, and high concentrations of this cytokine act as a chemoattractant for Th2 cells [154,155]. There is growing evidence that cells such as Th1, neutrophils, macrophages and natural killer cells (NK) express little ST2 in physiological conditions [156]. Yet, after priming with IL-12 in case of infection, the expression of the ST2 receptor, and thus susceptibility for IL-33 is highly increased [143,157]. This is emphasized by the ability of IL-33 to induce IFN-γ protein expression by aforementioned cells [158]. Subsequently, this mechanism is protective for the host as Bonilla et al. showed that IL-33 is needed for antiviral responses of CD8 cells in mice [159].

As briefly described above, there are several cytokines involved in the pathogenesis of osteoporosis. Yet, the involvement of IL-33 in osteoporotic patients has been studied well. Recently, IL-33 levels in the serum of 50 postmenopausal osteoporotic patients and 28 healthy postmenopausal control women were measured [160]. In postmenopausal osteoporotic women IL-33 was lower compared to controls and positively correlated respectively with serum levels of parathyroid hormone, while an inverse correlation was observed between IL-33 and C-terminal telopeptide of type 1 collagen levels. The authors suggest that IL-33 may represent an important bone-protecting cytokine which may hide therapeutic benefits for treating bone resorption.

### 2.3. Tumor Necrosis Factor-Alpha

Tumor necrosis factor-alpha was first described in 1975 by Carswell et al. for its cytotoxic activity to tumor cells via immune cells and thus was named TNF [161]. It is expressed as a type II transmembrane protein (mbTNFα) but can be cut to its soluble form (sTNFα) with increased biological activity [162]. The enzyme responsible for its cutting is TNF converting enzyme (TACE) or ADAM17 [163]. The membrane-bound mbTNFα has a 233 amino acid sequence, weighs 26 kDa and forms homotrimers [164]. This mbTNFα complex is cut to 51 kDa by TACE [165]. The main supply of TNF-α are macrophages and T-cells, yet many other cells such as B-cells, neutrophils, and endothelial cells have been described to produce TNF-α [165]. Targets for TNF-α include two type I transmembrane receptors, TNF receptor I (TNFR-I or CD120a) and TNF receptor II (TNFR-II or CD120b) [166]. Whereas TFNR-I is expressed on every cell except erythrocytes, TNFR-II is found only on endothelial and immune cells and can be activated by mbTNF [167].

The functional relevance is broad, and one prominent trait is the mediation of cell survival and pro-inflammatory response by TNFR-I via NF-κB and activator protein (AP)-1 [168]. Additionally, TNF-α instigates signaling pathways of cell death via Fas and Caspases [162,167]. For instance, this was demonstrated in in vitro ex vivo analyses of hematopoietic stem and progenitor cells of TNF-α *knockout* mice [169]. In a clinical study including 34 patients with at least 20% of total burn surface area, it was shown that systemic TNF levels correlated with burn severity and predicted a susceptibility to infection [170]. Just like in the case of IL-1, the determination of TNF-α levels can be very tricky because of a half-life of only 14 min [133]. Therefore, fast acquisition of blood samples to quantify TNF-α is imperative to use it as a potential biomarker. A German group reported significant levels of TNF-α and sTNF-α in blood samples taken 4, 12, and 24 h after admission to hospital as compared to control in patients with traumatic injury (*n* = 47) [171].

Adjacent to triggering the release of acute phase proteins after trauma, burns or infarction inter alia, TNF-α can initiate blood clotting [172]. Clinically, this can lead to a disseminated intravascular coagulation in case of severe inflammation like sepsis, cutting vital organs from blood perfusion, and thus driving them to failure [165,167]. To ensure necessary infiltration of immune cells to the local site of inflammation, e.g., in case of traumatic injury, vasodilation is essential [173]. Potent vasodilators are NO and prostaglandins like prostaglandin (PG)I_2_ or PGE_2_, which can be induced by TNF-α via iNOS and COX-2 upregulation [174]. In addition, expressions of adhesion molecules like E-selectin or ICAM-1 are upregulated by TNF-α aiding extravasation of monocytes and neutrophils in an endothelial cell model [175]. Yet, to effectively abolish bacterial infection, PMNLs use ROS as a means to destruct pathogens [176]. The essential protein for production of ROS is the nicotinamide adenine dinucleotide phosphate (NADPH)-oxidase [176]. In an endothelial cell model, TNF-α was shown to be a potent inductor of NADPH oxidase (NOX) proteins gp91^phox^, p22^phox^ and p67^phox^ that are needed for NADPH oxidase activation [177].

In the late eighties, the first in vivo studies with TNF-α antagonists were carried out showing promising results. In 1985, Beutler et al. showed that mice treated with anti-TNF-α serum had higher survival rates after LPS administration compared to control group mice [178]. Shortly after, Tracey et al. infused female baboons with anti-TNF-α antibodies, and injected them with a lethal dose of *Escherichia coli* (LD_100_) [173]. Baboons with antibodies were protected against shock, vital organ dysfunction, persistent stress hormone release and death as compared to control animals [173]. Yet, other studies with TNF-α *knockout* mice have provided evidence that, while animals may be protected against shock, they were far more susceptible to bacterial challenge [179]. Subsequent clinical studies with septic and shock patients have uncovered that there was no significant benefit for critically ill and septic patients treated with experimental TNF-α and sTNF-α antagonists [180]. It has to be considered that the used substances were not the known modern TNF-α antagonists. However, TNF-α antagonists such as etanercept, infliximab, or adalimumab proved to be a highly effective treatment for auto-inflammatory diseases like psoriasis, Crohn’s disease, or rheumatoid arthritis [168].

The role of the immune system in the onset of osteoporosis, a serious worldwide public health concern, is an area of current research. In a panel including 10 cytokines obtained from postmenopausal women, with either normal or low bone mineral density IL-23, IL-12, IL-4, IL-6, and also TNF-α levels were the most important differentiating cytokines [181]. However, no significant difference between the osteopenic and osteoporotic groups were found [181]. In general TNF-α suppresses osteoblasts activity at some stages of differentiation and stimulates osteoclast proliferation and differentiation [182]. Similar to IL-6, TNF-α can regulate bone metabolism through the endocrine way [183]. In a retrospective cohort analysis including a total of 199 rheumatoid arthritis patients, who were newly diagnosed with osteoporosis and receiving bisphosphonate changes in bone mineral density after one year were compared between patients treated with and without TNF inhibitors [184]. The therapy did not influence bone mineral density improvement in rheumatoid arthritis patients with osteoporosis receiving bisphosphonate [184]. However, although this data suggested that TNF inhibition cannot be considered as a preferred therapeutic option for increasing bone mineral density, conflictive findings have been reported showing that the use of bisphosphonate might be important to improve bone mineral density in patients with rheumatoid arthritis even under tight control [185]. Recently it was shown that altered T-cell activity and a different composition such as the CD14^+^CD16^+^ vs. CD14^+^CD16^-^ monocytes and priming of osteoclast precursors with increased macrophage colony-stimulating factor (M-CSF), receptor activator of NF-κB ligand (RANKL), and TNF-α levels in peripheral blood play a role in increased osteoclast formation and activity [186]. In summary, these findings may help the development of cytokine therapies for osteoporosis, and propose that the use of certain cytokine profiles as biomarkers for osteoporosis risk factors, may quantify the progress of therapies.

### 2.4. Interleukin-10

In 1989, IL-10 was first described by Fiorentino et al. as a cytokine synthesis inhibitory factor (CSIF) [187]. It is made up as a homodimer with each unit having a 178 amino acid sequence [188]. Interestingly, IL-10 is one of few anti-inflammatory cytokines next to IL-2, TGF, and the more recently discovered IL-25, IL-35, and IL-37 [189].

Biologically, IL-10 is usually found as a dimer and shares some structural and functional properties of interferon (IFN)-γ [190]. It is produced by almost all leukocytes including macrophages, dendritic cells, neutrophils, NK cells, B-cells, and CD8^+^ T-cells, however, CD4^+^ T-cells are the major producers [19,191]. For instance, FoxP3^+^ regulatory CD4^+^ T-cells (Tregs, thymus, and periphery-derived) and Foxp3^-^ regulatory CD4^+^ T-cells (Tr1 cells) attenuate T-cells and Th17 cell response in particular via IL-10 [19,192]. This review, however, will focus on the contribution of the cells of innate immunity.

Interestingly, some viruses like Epstein–Barr or Human Cytomegalovirus among others produce IL-10 homologs, which are almost identical to human IL-10 [193]. The receptor responsible for downstream signaling of IL-10 is the IL-10 receptor (IL10R), which is made up of dimers IL10R1 and IL10R2 [194]. The former is an IL-10 specific receptor and the primary binding site for IL-10, while the latter enhances the affinity of IL-10 to bind to IL10R1 [194]. In fact, IL10R2 cannot associate with IL-10 independently, and is expressed on many tissue cells [195], while IL10R1 is mostly expressed on immune cells such as T-cells [192], neutrophils upon LPS administration in vitro [196] or monocytes in a LPS endotoxemia mouse model [197]. Nevertheless, IL10R2 is a co-ligand to many other molecules like IL-22, IL-26, or IL-29, thereby playing a role in various biological pathways [193,198].

Downstream mediators of the IL-10 receptor are mainly STAT molecules and Janus Kinases (JAK) [199]. As a matter of fact, IL-10R1 is associated with JAK1 and IL10R2 with Tyk2 [19,200]. After activation by JAK, STAT dimer molecules like STAT1 and STAT5 in cytoplasm undergo a conformational switch, relocate to the nucleus and bind to DNA as transcription factors to IL10-responsive genes [201,202]. The number of genes regulated by IL-10 is numbering up to thousands with new genes discovered each year [19,203].

The biological effects of IL-10 on innate immune cells suppress the release of immune mediators, antigen expression and phagocytosis [193]. Indeed, in vitro inflammation models show that IL-10 prevents PMNL activation and TNF-α as well as IL-8 release after LPS administration [204]. Studies with human umbilical vein epithelial cells (HUVEC) display that IL-10 attenuates TNF-α induced ROS production, ICAM-1 expression, and leukocyte adhesion to HUVEC [205]. Recent research of monocyte models in vitro designate upregulation of ubiquitin ligases by IL-10 as the mechanism to sequestrate major histocompatibility complex (MHC) complexes and thus inhibit antigen presentation by antigen presenting cells (APCs) [206]. The overall influence on chemotaxis of monocytes is, however, rather low [207]. What is more, IL-10 *knockout* mice suffer from cardiac and vascular dysfunction due to an upsurge of COX-2 activity and production of prostaglandins indicating an important role in the suppression of COX by IL-10 [208]. Additionally, phagocytic cells are protected against complement lysis infused by an anti-MHC antibody or binding of zymosan when administered IL-10 compared to control cells in vitro [209]. Nevertheless, IL-10 plays an important role in suppressing inflammation in mucosa cells evident by IL-10 *knockout* mice that will develop severe colitis [210]. To limit its own properties, IL-10R activation also triggers the transcription of suppressor of cytokine signaling (SOCS)3, thus limiting its own release [211]. Albeit, systemic levels of IL-10 were significantly increased in patients with severe sepsis and linked to mortality as compared to patients with moderate sepsis [212]. A smaller study comparing 16 septic shock patients with 11 shock patients without sepsis supports the predictive value of systemic IL-10 levels in the first days after admission [213]. In case of trauma, a Swiss study reported elevated systemic IL-10 levels in patients with injury (*n* = 417) and a correlation of IL-10 levels to the severity of the injury as compared to healthy volunteers [214]. The development of sepsis in trauma patients was also linked to elevated systemic IL-10 levels on admission in an American study (*n* = 66) [215]. With regard to bone biology, loss of IL-10 exacerbated early Type-1 diabetes-induced bone loss [216]. Serum IL-10 levels in systemic lupus erythematosus patients with osteonecrosis were higher than that in those without osteonecrosis [217].

### 2.5. Interleukin-8

Interleukin-8 was first observed for its trait as a chemoattractant for granulocytes, primarily neutrophils in vitro [218]. It is sometimes called chemokine (C-X-C motif) ligand 8 or CXCL-8 and encoded by the CXCL-8 gene [219]. Through transfected cell culture models, NF-κB and JNK, as well as AP-1, have been identified as vital pathways for inducible IL-8 expression [220]. Every cell with TLR can produce and secrete IL-8 including macrophages and smooth muscle cells [221], while endothelial cells accumulate IL-8 in vesicles known as Weibel–Palade bodies [222]. Indeed, IL-8 is translated as a 99 amino acid long precursor peptide and cleaved into two active isoforms; one being 77 amino acids long and secreted by endothelial cells in cell culture [223]. While the other has a 72 amino acid sequence and is produced by monocytes and other leukocytes [224].

The main targets for IL-8 are G-protein coupled receptors CXCR1 and CXCR2, though the latter has a weaker affinity for IL-8 [225]. Furthermore, IL-8 guides neutrophils to the direction of inflammation (chemotaxis), which is evident in increased concentrations of this cytokine in lungs of patients with ARDS [226]. However, high IL-8 levels are not correlated with the probability of development of ARDS [227]. Additionally, IL-8 does not activate NAPDH oxidase directly with in vitro, yet, it enhances the respiratory burst activity by the recruitment of NAPDH oxidase components, N-formyl-methionyl-leucyl-phenylalanine (fMLP) receptor, and P-selectin ligands into lipid drafts [228]. In vitro studies with colon cancer cells transfected with IL-8 cDNA displayed a significant rise in cell proliferation, migration, and invasion by these cells [229]. This is supported by earlier in vitro investigations with HUVEC where recombinant IL-8 induced endothelial cell proliferation and capillary tube organization [230]. In conclusion, IL-8 is a very potent trigger to cell migration and proliferation, and thus should always be considered in inflammation models. A study analyzing systemic IL-8 levels for 60 days after a burn injury in children (*n* = 468) provided interesting insights [231]. The IL-8 levels correlated with the percent of burned total body surface area and were predictive for multiple organ failure and mortality [231].

It has to be considered that systemic IL-8 levels do not only provide prognostic value by reading the absolute levels, but rather implicate the duration of sustained high IL-8 levels for diagnosis. For instance, in a study with 27 patients, those with severe sepsis (*n* = 17) presented high IL-8 plasma levels steadily for 24 h after admission, whereas those with uncomplicated sepsis (*n* = 10) did not [232]. Furthermore, a smaller study with 24 subjects with traumatic brain injury (TBI) linked elevated systemic IL-8 levels upon admission to the worsened outcome [233]. This was first reported in a similar Croatian study with 20 TBI patients, with elevated IL-8 plasma levels in the non-survivor group [234].

Postmenopausal osteoporosis is characterized by rapid bone loss and IL-8 has been implicated among other pro-inflammatory cytokines to play a role in bone remodeling. There was a significant IL-8 increase in post-menopausal women with osteoporosis and bone loss [235]. Atorvastatin, which is known for its pleiotropic effects on bone tissue, decreased IL-8 levels and bone loss of rats subjected to glucocorticoid-induced osteoporosis [236]. RANKL-expressing neutrophils are increased in male patients with Chronic obstructive pulmonary disease (COPD), and furthermore, associated with bone mineral density and lung function, suggesting that these cells play a role in osteoclastogenesis in COPD [237]. Plasma levels of IL-8 were increased in COPD patients and correlated with RANKL expression by neutrophils [237].

### 2.6. Limitations

This review only provides a short overview of selected cytokines that are important for inflammatory reactions of the body. However, one should note that hundreds of other cytokines, hormones, and proteins mediate the immune dysregulation as seen in many patients with inflammatory states like sepsis or after trauma. A more comprehensive overview about immune dysregulation in shock/sepsis is given by Angus and van der Poll, as well as Rittirsch et al. [238,239]. Also, large randomized controlled trials or meta-analysis for clinical value of the mentioned cytokines are still missing. The overall data is still too weak to give a definite clinical evaluation.

## 3. Materials and Methods

For this review, existing literature was screened through Pubmed and Google Scholar. After careful consideration, cytokines IL-6, IL-1, IL-33, TNF-α, IL-10, and IL-8 were chosen as focus points for further research. We used respective names of the mentioned cytokines followed by terms “shock”, “inflammation”, “trauma”, “severe injury”, “immune dysregulation”, “osteoporosis”, “inflammasome”, and “critical illness” in various combinations. For each cytokine, we briefly described the biology by using the search engines described further above. Then, a selection of clinical studies for value as biomarkers is presented. We did not limit the time frame although more recent studies were favored.

## 4. Conclusions

New insights into molecular mechanisms provide a new perspective for finding appropriate biomarkers that may be helpful to predict severe or complicated cases upon clinical presentation. The constant discovery of, not only cytokines but other proteins, as well as receptors will pave the way for future diagnostics. The aforementioned cytokines are among the best studied. However, one should note that except for IL-6, none has really found its way from bench to bedside. Interestingly, they may be promising in the experimental environment, but further clinical research is needed. 

**Table 1:** Brief overview of cytokines that are involved in osteoporosis. ↑: upregulation.

Species	Study	Cell Type/Organ	Major Finding	Reference
**IL (Interleukin)-6**				
human	in vivo	bone	IL-6 is a predictor of postmenopausal bone loss	[240]
	meta-analysis	bone	GG genotype of IL-6-634C/G polymorphism seems to play a role in reducing bone mineral density	[47]
	meta-analysis	bone	IL-6-634C/G and IL-6-174G/C polymorphisms lead to modest effects on bone mineral density	[48]
	meta-analysis	bone	CC genotype of IL-6 G-174C polymorphism may be associated with high bone mineral density at femoral neck and distal radius and decreased risk of osteoporosis in the Caucasian population	[49]
	in vivo	bone	IL-6 G-174C promoter polymorphism may be a genetic marker for bone loss and wrist fracture among older women	[241]
	meta-analysis	bone	IL6-174 G/C gene polymorphism positively correlated with osteoporosis risk	[242,243]
	in vivo	bone	Variation within the low levels of IL-6 predicts bone loss and resorption	[244]
	in vitro	whole blood cells	Increased IL-6 production by whole blood cells from postmenopausal women with osteoporosis compared to controls	[245]
	in vivo	bone	IL-6 is upregulated in postmenopausal women with low bone mineral density compared to postmenopausal women with normal bone mineral density	[181]
	Chronic obstructive pulmonary disease (COPD) patients	bone	RANKL (Receptor activator of NF-κB ligand)-expressing neutrophils correlate negatively with bone marrow density. Plasma levels of IL-6 are increased in COPD patients and correlate with RANKL expression by neutrophils	[237]
mouse	in vivo	osteoclasts	IL-6 mediates stimulation of osteoclastogenesis after estrogen loss	[246]
**IL-1**				
human	in vitro	whole blood cells	Increased IL-1 beta production by whole blood cells from postmenopausal women with osteoporosis compared to controls	[245]
	COPD patients	bone	RANKL-expressing neutrophils correlate negatively with bone marrow density. Plasma levels of IL-1 beta are increased in COPD patients and correlate with RANKL expression by neutrophils	[237]
	in vivo	bone	IL-1β (-511C/T) polymorphism is associated with pathogenesis of osteoporosis in postmenopausal women	[247]
	in vivo	bone	Serum IL-1β significantly higher in women with osteoporosis than controls	[248]
	in vivo	bone	Serum IL-1 is significantly reduced after treatment of postmenopausal with calcitriol	[249]
	in vitro	osteoblasts	IL-1beta and Tumor necrosis factor-alpha (TNF-α) regulate osteoblast cell number by up-regulating the Fas-mediated apoptosis of osteoblasts	[250]
rat	in vivo	bone	Interleukin-1 receptor antagonist decreases bone loss and bone resorption in a rat model of postmenopausal osteoporosis	[251]
**IL-33**				
human	in vivo	bone	IL-33 levels in postmenopausal women significantly lower compared to healthy controls, positively correlated with serum parathyroid hormone and inverse correlated with C-terminal telopeptide of type 1 collagen	[160]
**TNF-α**				
human	in vitro	whole blood cells	Increased TNF-α production by whole blood cells from postmenopausal women with osteoporosis compared to controls	[245]
	in vivo	bone	TNF-α is upregulated in postmenopausal women with low bone mineral density compared to postmenopausal women with normal bone mineral density	[181]
	in vivo + in vitro	osteoclasts	Estrogen deficiency → TNF-α and RANKL ↑ → osteoclast formation and number of osteoclast precursors ↑	[252]
	in vivo	bone	Association between TNF-α-308G>A polymorphism and postmenopausal osteoporosis	[253]
	in vivo	bone	Serum TNF-α is significantly reduced after treatment of postmenopausal with calcitriol	[249]
	in vivo in vitro		TNF-α increased in postmenopausal women with osteoporosis and highly correlated with the RANK and estrogen levels TNF-α synergistically promotes RANKL-induced osteoclasts formation through activation of Phosphoinositide 3-kinases (PI3K)/Akt signaling	[254]
	in vitro	mesenchymal stem cells (MSC)	TNF-α suppresses osteogenic differentiation of MSCs by accelerating P2Y2 receptor in estrogen-deficiency induced osteoporosis	[255]
	in vitro	osteoblasts	IL-1beta and TNF-α regulate osteoblast cell number by up-regulating the Fas-mediated apoptosis of osteoblasts	[250]
mouse	in vivo	bone marrow-derived mesenchymal stem cells (BMMSCs)	TNF-α inhibits Forkhead box protein O1 (FoxO1) and thereby aggravates oxidative damage in BMMSCs during osteoporosis	[256]
rat	in vitro	bone cultures of fetal rat calvariae	TNF-α causes osteoclastic bone resorption and inhibits bone collagen synthesis	[257]
**IL-10**				
human	patients with systemic lupus erythematosus	bone	IL-10 level is elevated in systemic lupus erythematosus patients with osteoporosis	[217]
	in vivo	bone	IL-10 gene-597 C>A polymorphism is associated with higher risk for osteoporosis	[258]
	in vivo	bone	Lower levels of IL-10 in postmenopausal women with osteoporosis	[259,260]
	in vivo	bone	Association between IL10-1082G>A polymorphism and postmenopausal osteoporosis	[253]
mouse	in vitro	RAW264.7 monocytes	IL-10 directly inhibits osteoclastogenesis is by suppressing Nuclear factor of activated T-cells, cytoplasmic 1 activity	[261]
	in vivo	bone	IL-10 is important for promoting osteoblast maturation and reducing bone loss during early stages of type-1 diabetes	[216]
	in vivo	bone	IL-10^−/−^ mice develop the reduced bone mass, increased mechanical fragility, and suppressed bone formation (hallmarks of osteoporosis)	[262]
**IL-8**				
human	in vivo	bone	IL-8 significantly increase in post-menopausal women with osteoporosis and bone loss	[235]
	COPD patients	bone	RANKL-expressing neutrophils correlate negatively with bone marrow density, plasma levels of IL-8 are increased in COPD patients and correlate with RANKL expression by neutrophils	[237]
	in vitro	osteoblasts	IL-8 may contribute to osteoporosis in rheumatoid arthritis by enhanced osteoblast-mediated osteoclastogenesis (partly via IL-6 production)	[263]
rats	in vivo	bone	IL-8 and bone loss reduction after atorvastatin treatment of rats with glucocorticoid-induced osteoporosis	[236]

## Abbreviations

ADAM	A disintegrin and metalloproteinase
AP	Activator protein
APC	Antigen presenting cells
ARDS	Acute respiratory distress syndrome
ASC	Apoptosis-associated speck-like protein containing a CARD
ATP	Adenosine triphosphate
CARD	Caspase activation and recruitment domain
CD	Cluster of differentiation
COPD	Chronic obstructive pulmonary disease
COX	Cyclooxygenase
CRP	C-reactive protein
CSIF	Cytokine synthesis inhibitory factor
DAMP	Damage-associated molecular patterns
DNA	Deoxyribonucleic acid
fMLP	Formyl-methionyl-leucyl-phenylalanine
FoxO1	Forkhead box protein O1
gp	Glycoprotein
HMGB1	High-mobility group box 1
HSP	Heat shock proteins
ICAM	Intercellular cell adhesion molecules
IFN	Interferon
IL	Interleukin
ILC	Innate lymphoid cells
IL-1R	Interleukin-1 receptor
iNOS	Inducible nitric oxide synthase
kDa	Kilo Dalton
LPS	Lipopolysaccharide
LRR	Leucine-rich repeat
MAP	Mitogen activated protein
mbIL6R	membrane-bound IL-6 receptor
MBL	Mannose-binding lectin
MCP	Monocyte chemotactic protein
M-CSF	Macrophage-colony stimulating factor
MHC	Major histocompatibility complex
MODS	Multi organ dysfunction syndrome
MyD88	Myeloid differentiation primary response 88
NADPH	Nicotinamide adenine dinucleotide phosphate
NF-HEV	Nuclear factor from high endothelial venules
NF-κB	nuclear factor ‘kappa-light-chain-enhancer’ of activated B-cells
NLR	NOD-Like Receptors
NLRP	NOD-, LRR- and pyrin domaine-containing protein
NO	Nitric oxide
NOD	Nucleotide-binding oligomerization domain
NPC	Nasopharyngeal carcinoma
PAMP	Pathogen-associated molecular patterns
PBMC	Peripheral blood mononuclear cells
PG	Prostaglandin
PGN	Peptidoglycan
PI3K	Phosphoinositide 3-kinases
PLA	Phospholipase A
PMNL	Polymorphonuclear leukocytes
PRR	Pattern recognition receptors
R, r	Receptor
RANKL	Receptor activator of NF-κB ligand
ROS	Reactive oxygen species
s	Soluble
sIL6R	IL-6 receptor
STAT	Signal transducer and activator of transcription
TACE	TNF converting enzyme
TBI	Traumatic brain injury
Th	T helper cells
TLR	Toll-like receptors
TNF-α	Tumor necrosis factor-alpha
VCAM	Vascular cell adhesion molecule
Wnt	Wingless-related integration site
